# Critical hypertension in trauma patients following prehospital emergency anaesthesia: a multi-centre retrospective observational study

**DOI:** 10.1186/s13049-023-01167-w

**Published:** 2023-12-20

**Authors:** Liam Sagi, James Price, Kate Lachowycz, Zachary Starr, Rob Major, Chris Keeliher, Benjamin Finbow, Sarah McLachlan, Lyle Moncur, Alistair Steel, Peter B. Sherren, Ed B G Barnard

**Affiliations:** 1 Department of Research, Audit, Innovation, and Development, East Anglian Air Ambulance, Norwich, UK; 2 Emergency Department, Cambridge University Hospitals NHS Foundation Trust, Cambridge, UK; 3 Essex and Herts Air Ambulance, Earls Colne, UK; 4 Anglia Ruskin University, Chelmsford, UK; 5 Magpas Air Ambulance, Huntingdon, UK; 6 Department of Critical Care Medicine, Guy’s and St Thomas’ NHS Foundation Trust, London, UK; 7 Academic Department of Military Emergency Medicine, Royal Centre for Defence Medicine (Research & Clinical Innovation), Birmingham, UK

**Keywords:** Air ambulance, Anaesthesia, Blood pressure, Helicopter emergency medical service, Hypertension, Hypotension, Prehospital, Prehospital emergency care, Rapid sequence induction, Trauma

## Abstract

**Background:**

Critical hypertension in major trauma patients is associated with increased mortality. Prehospital emergency anaesthesia (PHEA) is performed for 10% of the most seriously injured patients. Optimising oxygenation, ventilation, and cerebral perfusion, whilst avoiding extreme haemodynamic fluctuations are the cornerstones of reducing secondary brain injury. The aim of this study was to report the differential determinants of post-PHEA critical hypertension in a large regional dataset of trauma patients across three Helicopter Emergency Medical Service (HEMS) organisations.

**Methods:**

A multi-centre retrospective observational study of consecutive adult trauma patients undergoing PHEA across three HEMS in the United Kingdom; 2015–2022. Critical hypertension was defined as a new systolic blood pressure (SBP) > 180mmHg within 10 min of induction of anaesthesia, or > 10% increase if the baseline SBP was > 180mmHg prior to induction. Purposeful logistical regression was used to explore variables associated with post-PHEA critical hypertension in a multivariable model. Data are reported as number (percentage), and odds ratio (OR) with 95% confidence interval (95%CI).

**Results:**

30,744 patients were attended by HEMS during the study period; 2161 received PHEA and 1355 patients were included in the final analysis. 161 (11.9%) patients had one or more new episode(s) of critical hypertension ≤ 10 min post-PHEA. Increasing age (compared with 16–34 years): 35–54 years (OR 1.76, 95%CI 1.03–3.06); 55–74 years (OR 2.00, 95%CI 1.19–3.44); ≥75 years (OR 2.38, 95%CI 1.31–4.35), pre-PHEA Glasgow Coma Scale (GCS) motor score four (OR 2.17, 95%CI 1.19–4.01) and five (OR 2.82, 95%CI 1.60–7.09), patients with a pre-PHEA SBP > 140mmHg (OR 6.72, 95%CI 4.38–10.54), and more than one intubation attempt (OR 1.75, 95%CI 1.01–2.96) were associated with post-PHEA critical hypertension.

**Conclusion:**

Delivery of PHEA to seriously injured trauma patients risks haemodynamic fluctuation. In adult trauma patients undergoing PHEA, 11.9% of patients experienced post-PHEA critical hypertension. Increasing age, pre-PHEA GCS motor score four and five, patients with a pre-PHEA SBP > 140mmHg, and more than intubation attempt were independently associated with post-PHEA critical hypertension.

## Background

Timely delivery of prehospital emergency anaesthesia (PHEA) to the seriously injured trauma patient with airway compromise or ventilatory failure is performed to reduce significant and potentially preventable morbidity and mortality [1, 2]. Every year in the United Kingdom (UK), approximately 10% of major trauma patients undergo PHEA by physician-paramedic Helicopter Emergency Medical Services (HEMS) [3]. Blunt traumatic head injury is the predominant injury pattern, of which one third of patients have concomitant thoracic and abdominal injury [2, 4–7]. The aim of PHEA is to secure a definitive airway, optimise ventilation, and commence early neuroprotection.

The ideal PHEA avoids negative physiological changes, reduces secondary insult through optimised oxygenation and ventilation, provides neuroprotection, and should be balanced with effective haemorrhage resuscitation strategies where required. Physiological derangement is deleterious - a single episode of hypotension significantly increases mortality in traumatic brain injury (TBI) [8, 9]. Hypertension is also associated with a greater mortality risk, especially in TBI patients with a systolic blood pressure (SBP) > 180mmHg [10–13]. In addition, hypertension may cause myocardial ischaemia, harmful increases in intracranial pressure (ICP), and increase the risk of aneurysmal rupture [10, 14–17].

Traditional PHEA induction drug regimes have been superseded by fentanyl, ketamine, and rocuronium, in response to data demonstrating unwarranted post-intubation hypertension [18]. The addition of fentanyl attenuates the haemodynamic response to ketamine and laryngoscopy, and the associated cardiovascular and potential neurological complications [18]. Since the introduction of this regime, research has predominantly focussed on post-induction *hypo*tension [7, 17, 19–24]. However, current prehospital TBI management guidelines place new emphasis on the recognition and management of hypertension in the prehospital phase [13]. Despite emerging evidence of the negative effects of hypertension in critically injured trauma patients, there is a paucity of published evidence in this area [10–12]. The aim of this study was to report the incidence and differential determinants of post-PHEA critical hypertension in prehospital trauma patients attended by HEMS.

## Methods

### Setting

This study was performed in the East of England, a mixed urban and rural geographical area of 20,000 km^2^ with a population of approximately 6.4 million people [25]. HEMS provide prehospital critical care to the patients of the East of England Ambulance Service NHS Trust, and operate from five bases: two East Anglian Air Ambulance (EAAA), two Essex & Herts Air Ambulance (EHAAT), and one Magpas Air Ambulance (Magpas). All teams utilise a combination of rotary wing aircraft and ground-based rapid response vehicles dependant on time of day, patient location, and aviation restrictions.

A physician-critical care paramedic (CCP) model is employed by all three HEMS. Physicians are predominantly emergency medicine (EM) or anaesthetic specialists at the level of consultant or senior registrar (at least five years post-graduation), with a minimum of six-months in-hospital anaesthesia training. CCPs have at least three years’ post-registration experience. Physicians and CCPs undergo additional specialist critical care training followed by supervised practice and formative assessment by a prehospital consultant (physicians) or senior CCP prior to autonomous practice. A programme of continued training supports skill retention.

To standardise practice across the region, all three HEMS use a shared PHEA standard operating procedure (SOP). This SOP includes a recommended PHEA induction regime: ketamine (1–2 mg kg^− 1^), rocuronium (1 mg kg^− 1^), ± fentanyl (1–3 mcg kg^− 1^) at the discretion of the attending clinician to attenuate the hypertensive response to laryngoscopy; modified according to patient age, weight, haemodynamics, and suspected injury [18]. Maintenance of anaesthesia is recommended as either infusion: ketamine (1 mg/kg/hr); propofol 1% (2-5 mg/kg/hr) or as bolus: ketamine (up to 0.25 mg kg^− 1^); midazolam (up to 0.025 mg kg^− 1^ ); fentanyl (0.5 mcg kg^− 1^ ) adjusted according to patient physiology. Laryngoscopy is typically performed 45–60 s after administration of rocuronium. Direct laryngoscopy (DL) was the primary method of airway visualisation until 2017 when videolaryngoscopy (VL) (McGrath® Video Laryngoscope, Aircraft Medical, Edinburgh, UK) was introduced for use at the preference of the intubating clinician at EAAA and Magpas. All HEMS utilise a ‘challenge and response’ pre-PHEA checklist to optimise physiology and conditions prior to induction [3]. Positive pressure ventilation (6–8 ml kg^− 1^ ideal body weight) with a positive end-expiratory pressure (PEEP) of 5 cmH_2_O, and rate to achieve normocapnia are standard practice. HEMSbase (MedicOne Systems Ltd, UK) is the electronic medical record software used by each HEMS to capture data and patient information.

### Inclusion criteria

In this retrospective observational study, we included a consecutive sample of all trauma patients aged ≥ 16 years attended by EAAA and EHAAT between 1st January 2015 and 31st December 2022 and Magpas between 1st November 2015 to 31st December 2022 (owing to the later adoption of the common electronic medical record system) who received a PHEA, defined as a drug-assisted intubation.

### Exclusion criteria

Patients who were intubated in cardiac arrest, patients undergoing inter-facility transfer, patients intubated by a non-HEMS team provider, and patients with an injury mechanism other than trauma (injury through transfer of kinetic energy): hanging, drowning, overdose, asphyxia, burns, electrocution, and any other similar non-traumatic mechanism were excluded. Patient records were reviewed by one of the study authors to identify exclusions. Patients without any pre- or post-PHEA SBP readings were excluded following interrogation.

### Data collection

Routinely collected pseudonymised patient data from the electronic medical record were extracted and collated in a single data spreadsheet (Microsoft® Excel for Mac, v16.45) and stored in an organisational secure data environment.

Extracted data items included: demographics (age, sex, weight), suspected injury pattern, mechanism and type of injury (blunt or penetrating), Glasgow Coma Scale total (GCS-T) score, Glasgow Coma Scale motor (GCS-M) score, PHEA indication, fluid administered pre-PHEA, intubation attempts, use of direct or video-laryngoscope, time interval from HEMS team arrival to PHEA (minutes), drug dosage: fentanyl (mcg kg^− 1^), ketamine (mg kg^− 1^), and rocuronium (mg kg^− 1^).

Physiological data recorded routinely by time-calibrated monitors (EAAA – X Series®, ZOLL Medical Corporation, Runcorn, UK; EHAAT & Magpas – Tempus Pro, Philips Electronics UK Ltd, Farnborough, UK) and automatically uploaded to HEMSbase at two-minute (EAAA, EHAAT) or three-minute (Magpas) intervals were collected and interrogated. Manual interrogation was undertaken to record the following pre-PHEA physiological variables based on the closest time-point preceding the recorded PHEA induction time: heart rate (HR), respiratory rate (RR), systolic blood pressure (SBP), and diastolic blood pressure (DBP). Post-PHEA data (HR, SBP, and DBP) were documented at two-minute intervals (or closest time point) up to ten minutes. Apparently erroneous data were reviewed by two data interrogators and excluded if deemed explicitly erroneous.

PHEA induction drug doses were collected; fentanyl (mcg), ketamine (mg), and rocuronium (mg), and divided by the estimated patient weight (kg). These were rounded to the nearest integer and expressed as a rounded dose per kg, with 0,1,2,3 (mcg kg^− 1^) categories used for fentanyl and 0,1,2 (mg kg^− 1^) categories used for ketamine and rocuronium. Patients who did not receive the drug were coded as 0, those receiving > 0.5 per kg but < 1.5 per kg were rounded to 1. Patients who received greater than the maximum dose were included in the maximum category for the drug.

### Primary outcome

The primary outcome was post-PHEA critical hypertension defined as at least one new episode of SBP > 180mmHg ≤ 10 min of induction, or a > 10% increase if SBP was > 180mmHg pre-PHEA [10, 13].

### Data analysis

Data management and statistical analyses were performed in R statistical programming language (R Core Team [2018]; R: A language and environment for statistical computing [R Foundation for Statistical Computing, Vienna, Austria]). A pre-defined significance value of *p* < 0.05 was used throughout. The sample characteristics were described using number (percentage) for categorical variables and mean (± standard deviation (SD)) or median [interquartile range (IQR)] for continuous variables as appropriate.

Logistic regression was used to test the association between the variables and post-PHEA hypertension and coded as a binary outcome (1 = hypertension, 0 = no hypertension). A purposeful model was used to select variables [26]. First, each variable was tested individually to document its unadjusted association with the outcome. A multivariable model was then built using variables with a *p*-value of < 0.25 from the univariate analysis or of known clinical importance. Backward stepwise elimination was used to find a reduced model that best fits the data, defined at the highest likelihood ratio test for goodness of fit. The final model was assessed to ensure it met the assumptions necessary for logistic regression: no multicollinearity in the variables, linear relationships in the logit of the outcomes, and no unduly influential values. Plausible interactions between variables were tested by including interaction variables in the model and assessing significance and goodness of fit.

For variables grouped in categories, the group containing the most cases was used as the baseline reference in the regression model. Patients were divided into four age groups: 16–34 years; 35–54 years; 55–74 years; 75 + years. Pre-PHEA SBP was grouped as: Low (< 90), Mid (90–140), High (141–180), Critical (> 180) mmHg; heart rate was grouped as: Low (< 60), Mid (60–100), High (> 100) beats/minute; and respiratory rate was grouped as: Low (< 10), Mid [10–25], High (> 25) breaths/minute [27]. For all drug dosage categories, 1 mg/mcg kg^− 1^ was used as the reference group.

### Ethical review

Ethical approval for the study was granted by Allied Health, Nursing & Midwifery and Medicine School Research Ethics Panel at Anglia Ruskin University (ETH2223-3743). The STROBE (Strengthening the Reporting of Observational studies in Epidemiology) reporting guideline was followed [28].

## Results

**Fig. 1:**
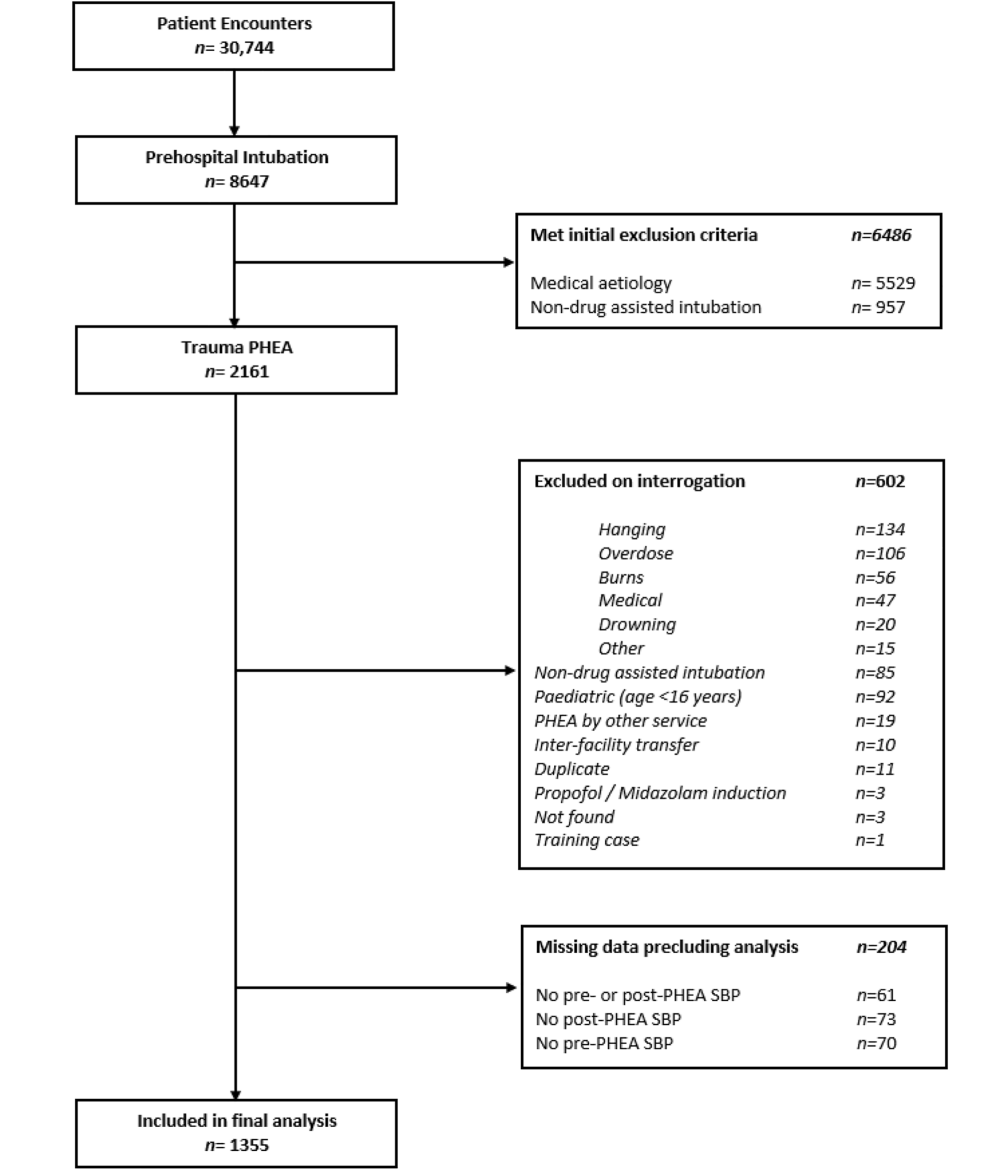
Study flow diagram of adult trauma patients who underwent PHEA in the East of England (2015–2022). Cases excluded on interrogation based on mechanism of injury with *n* = < 10 are grouped to ‘Other’ to protect patient confidentiality and include: smoke inhalation, asphyxiation, electrocution and hypothermia. ‘Training case’ refers to a fictional patient record that was created for the purpose of training and education. PHEA prehospital emergency anaesthesia, SBP systolic blood pressure

During the study period, 30,744 patients were attended by HEMS. *N =* 2161 trauma patients underwent PHEA, of which 1355 were included in the final analysis, per protocol: EHAAT *n =* 576 (42.7%), EAAA *n =* 545 (41.7%), Magpas *n =* 234 (15.6%), Fig. 1.

*N* = 1023 (75.4%) were male and *n* = 462 (34.1%) were aged 16–34 years. Blunt trauma accounted for *n =* 1306 (96.4%) of injury patterns, with suspected isolated head injury most frequently observed (*n =* 706, 52.1%). In addition to a suspected head injury, *n =* 464 patients (34.2%) had a concomitant thoracic and/or abdominal injury. The predominant mechanism of injury was ‘transport’ (*n =* 737, 54.4%). Intravenous crystalloid was administered by the ambulance service prior to HEMS arrival for *n =* 367 (27.1%) of patients, Table 1.

**Table 1:** Patient characteristics, physiological variables and PHEA characteristics in adult trauma patients who underwent PHEA in the East of England; 2015–2022

Variable		Total (*n* = 1355)
**Sex / n (%)**	Male	1023 (75.5%)
	Female	332 (24.5%)
**Age group / n (%)**	16–34	462 (34.1%)
	35–54	384 (28.3%)
	55–74	336 (24.8%)
	75+	173 (12.8%)
**Estimated patient weight / kg, median [IQR]**		80 [70–80]
**Glasgow Coma Scale (GCS) score / median [IQR]**		7 [4–11]
**GCS motor score / n (%)**	1	310 (22.9%)
	2	99 (7.3%)
	3	142 (10.5%)
	4	243 (17.9%)
	5	293 (21.6%)
	6	268 (19.8%)
**Suspected injury pattern / n (%)**	Isolated head injury	706 (52.1%)
	Head injury + thorax/abdomen	464 (34.2%)
	No head injury	185 (13.7%)
**Trauma type / n (%)**	Blunt	1306 (96.4%)
	Penetrating	49 (3.6%)
**Mechanism / n (%)**	Transport	737 (54.4%)
	Accidental Injury	442 (32.6%)
	Assault	69 (5.1%)
	Self-harm	66 (4.9%)
	Sport/leisure	41 (3.0%)
**Pre-PHEA Shock index / median [IQR]**		0.69 [0.54–0.93]
**Pre-PHEA SBP / mmHg, n (%)**	Low (< 90)	132 (9.7%)
	Mid (90–140)	688 (50.8%)
	High (141–180) Critical (> 180)	417 (30.8%) 118 (8.7%)
**Pre-PHEA HR / beats/minute, n (%)**	Low (< 60)	120 (8.9%)
	Mid (60–100)	669 (49.4%)
	High (> 100)	563 (41.5%)
	NA	3 (0.2%)
**Pre-PHEA RR / breaths/minute, n (%)**	Low (< 10)	74 (5.5%)
	Mid (10–25)	622 (45.9%)
	High (> 25)	276 (20.4%)
	NA	383 (28.3%)
**Fentanyl dose (mcg kg** ^**− 1**^ **) / n (%)**	0	446 (32.9%)
	1	398 (29.4%)
	2	120 (8.9%)
	3	391 (28.9%)
**Ketamine dose (mg kg** ^**− 1**^ **) / n (%)**	0	76 (5.6%)
	1	733 (54.1%)
	2	546 (40.3%)
**Rocuronium dose (mg kg** ^**− 1**^ **) / n (%)**	0	35 (2.6%)
	1	1206 (89.0%)
	2	114 (8.4%)
**Indication for PHEA / n (%)**	Reduced consciousness	613 (45.2%)
	Airway obstruction/compromise	280 (20.7%)
	Agitated head injury	172 (12.7%)
	Ventilatory failure	165 (12.2%)
	Anticipated clinical course	102 (7.5%)
	Other	23 (1.7%)
**Arrival time to PHEA in minutes / median [IQR]**		24 [18–32]
**Intubation attempts / n (%)**	1 > 1	1231 (90.8%) 124 (9.2%)
**Blade type / n (%)**	Direct laryngoscope (DL) Video laryngoscope (VL) NA	1034 (76.3%) 319 (23.5%) 2 (0.2%)
**Pre-PHEA fluids / n (%)**	None	988 (72.9%)
	Fluids	367 (27.1%)

GCS Glasgow Coma Scale, PHEA prehospital emergency anaesthesia, RSI rapid sequence induction, HR heart rate, SBP systolic blood pressure, RR respiratory rate. The shock index was calculated as HR/SBP. ‘Arrival to PHEA’ is the time in minutes from the Helicopter Emergency Medical Service team arrival on scene until the time of PHEA. Pre-PHEA fluids are intravenous crystalloid administration by the ambulance service before arrival of HEMS

*N =* 161 (11.9%) patients had one or more new episode(s) of critical hypertension ≤ 10 min of PHEA, of which, *n =* 147 (91.3%) had a pre-PHEA SBP < 180mmHg. *N =* 14 (8.7%) were hypertensive (> 180mmHg) pre-PHEA and their SBP increased by > 10% post-PHEA. The most prevalent indication for PHEA was ‘reduced level of consciousness’, *n* = 613 (45.2%).

**Fig. 2:**
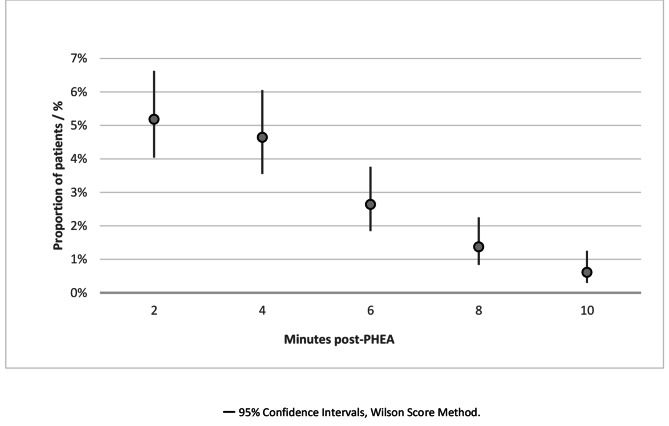
Point estimate chart showing the proportion of adult trauma patients who underwent PHEA in the East of England (2015–2022) with a new episode of critical hypertension (defined as a new SBP > 180 mmHg ≤ 10 min of induction, or a > 10% increase if SBP was > 180 mmHg pre-PHEA) at two-minute epochs within the first ten minutes following induction. PHEA prehospital emergency anaesthesia, SBP systolic blood pressure

Figure 2 shows the incidence of critical hypertension at two-minute epochs, defined as the first new episode of hypertension for each patient. The peak incidence of post-PHEA hypertension was at two minutes.

**Fig. 3:**
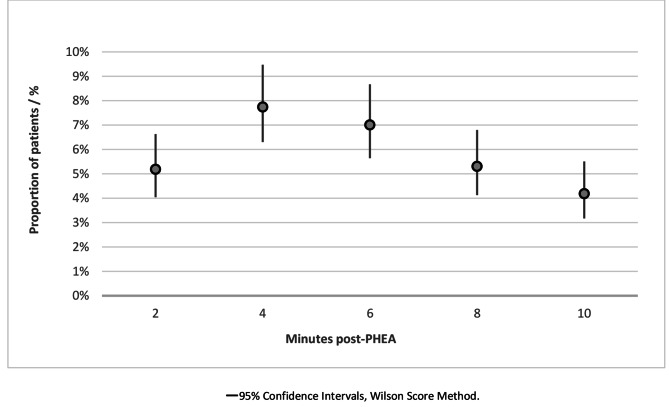
Point estimate chart showing the prevalence (cases at point in time) of critical hypertension (defined as a new SBP > 180 mmHg ≤ 10 min of induction, or a > 10% increase if SBP was > 180 mmHg pre-PHEA) at two-minute epochs within the first ten minutes following induction, in adult trauma patients who underwent PHEA in the East of England (2015–2022). PHEA prehospital emergency anaesthesia, SBP systolic blood pressure

Figure 3 shows the point prevalence of critical hypertension across the ten minutes, with a peak at four minutes, when *n =* 85, (7.7%) of patients had an episode of critical hypertension.

Table 2 shows the univariate association of the variables with the outcome.

**Table 2:** Univariate analysis: Association of variables with critical hypertension (defined as a new SBP > 180 mmHg ≤ 10 min of induction, or a > 10% increase if SBP was > 180 mmHg pre-PHEA) of adult trauma patients undergoing PHEA in the East of England, 2015 − 2002

Variable		Coefficient	*P*-value
**Sex**	Female Male	REF 0.177	0.383
**Age / years**	16–34 35–54 55–74 75+	REF 0.749 1.252 1.411	0.004* < 0.001* < 0.001*
**Estimated patient weight**		0.007	0.220*
**Glasgow Coma Scale score**		0.040	0.053*
**GCS motor score**	1 2 3 4 5 6	REF 0.158 0.255 0.698 0.879 0.355	0.700 0.470 0.013* 0.001* 0.220*
**Mechanism**	Transport Accidental Injury Assault Self-harm Sport/leisure	REF 0.649 0.528 < 0.001 0.722	< 0.001* 0.149* 1.000 0.096*
**Shock index (SI)**		-3.011	< 0.001*
**Pre-PHEA SBP / mmHg**	Mid (90–140) Low (< 90) High (141–180) Critical (> 180)	REF -1.186 1.986 0.983	0.106* < 0.001* 0.003*
**Pre-PHEA HR / beats/minute**	Mid (60–100) Low < 60) High (> 100)	REF 0.165 -0.910	0.531 < 0.001*
**Pre-PHEA RR / breaths/minute**	Mid (10–25) High (> 25) Low (< 10)	REF -0.261 -0.623	0.277 0.194*
**Fentanyl dose (mcg kg** ^**− 1**^ **)**	1 0 2 3	REF -0.695 -0.256 -0.063	0.002* 0.421 0.759
**Ketamine dose (mg kg** ^**− 1**^ **)**	1 0 2	REF -0.719 0.439	0.173* 0.010*
**Rocuronium dose (mg kg** ^**− 1**^ **)**	1 0 2	REF 0.628 -0.126	0.146* 0.692
**Indication for PHEA**	Reduced consciousness Airway obstruction/compromise Ventilatory failure Agitated head injury Anticipated clinical course Other	REF -0.286 -0.830 0.273 -0.104 0.353	0.219* 0.017* 0.253 0.753 0.531
**Arrival time to PHEA / minutes**		0.009	0.146*
**Total attempts**		0.499	0.006*
**Blade type**	Direct Laryngoscope Video Laryngoscope	REF 0.335	0.073*
**Pre-PHEA fluids**	None Fluids	REF -0.833	< 0.001*

* p < 0.25 (threshold for including in first iteration of multivariate model)

PHEA prehospital emergency anaesthesia, RSI rapid sequence induction, HR heart rate, SBP systolic blood pressure, RR respiratory rate. The shock index was calculated as HR/SBP. ‘Arrival to PHEA’ is the time in minutes from the Helicopter Emergency Medical Services (HEMS) team arrival on scene until the time of PHEA. Pre-PHEA fluids are intravenous crystalloid administration by the ambulance service before arrival of HEMS

### Primary outcome

Six variables were associated with post-PHEA critical hypertension: increasing age (≥ 35 years old, with the greatest effect size in those aged > 75 years old), patients with a GCS-M score of four or five on primary survey, a pre-PHEA SBP > 140mmHg, and more than one intubation attempt. In contrast, larger doses of fentanyl on induction (up to 2mcg kg^− 1^), a heart rate > 100 beats/minute, and administration of crystalloid by the ambulance service prior to HEMS arrival were variables associated with reduced incidence of post-PHEA critical hypertension, Table 3.

**Table 3:** Final multivariate model – adjusted association of variables with critical hypertension (defined as a new SBP > 180 mmHg ≤ 10 min of induction, or a > 10% increase if SBP was > 180 mmHg pre-PHEA) in adult trauma patients undergoing PHEA in the East of England, 2015–2022

Variable		Adjusted Odds ratio (95% CI)	*P*-value
**Age / years**	**16–34**	**REF**	
	35–54	1.76 (1.03–3.06)	0.039*
	55–74	2.00 (1.19–3.44)	0.010*
	75+	2.38 (1.31–4.35)	0.005**
**GCS motor score**	**1**	**REF**	
	2	1.04 (0.42–2.40)	0.923
	3	1.22 (0.57–2.54)	0.596
	4	2.17 (1.19–4.01)	0.012*
	5	2.82 (1.60–5.09)	< 0.001***
	6	1.79 (0.99–3.33)	0.064
**Pre-PHEA SBP / mmHg**	**Mid (90–140)**	**REF**	
	Low < 90)	0.36 (0.057–1.23)	0.166
	High (> 140)	6.72 (4.38–10.54)	< 0.001***
	Critical (> 180)	2.19 (1.05–4.39)	0.031*
**Pre-PHEA HR / beats/minute**	**Mid (60–100)**	**REF**	
	Low (< 60)	1.55 (0.85–2.72)	0.190
	High (> 100)	0.42 (0.27–0.64)	< 0.001***
**Fentanyl dose (mcg kg** ^**− 1**^ **)**	**1**	**REF**	
	2	0.47 (0.23–0.90)	0.029*
	3	0.67 (0.42–1.05)	0.083
	0	0.82 (0.50–1.34)	0.568
**Pre-PHEA fluids**	**None**	**REF**	
	Fluids	0.60 (0.36–0.97)	0.043*
**Intubation attempts**	**1**	**REF**	
	> 1	1.75 (1.01–2.96)	0.040*

***p < 0.001, ** p < 0.01, * p < 0.05

PHEA prehospital emergency anaesthesia, RSI rapid sequence induction, HR heart rate, SBP systolic blood pressure, RR respiratory rate. Pre-PHEA fluids are intravenous crystalloid administration by the ambulance service before arrival of Helicopter Emergency Medical Service

## Discussion

This study has demonstrated that more than one in ten patients who underwent PHEA for serious traumatic injury exhibited a new episode of critical hypertension within the first ten minutes of anaesthesia. Increasing age, pre-PHEA GCS-M score of four and five, patients with a pre-PHEA SBP > 140mmHg, and patients in whom more than one intubation attempt was performed were associated with post-PHEA critical hypertension. Larger fentanyl doses on induction (up to 2mcg kg^− 1^), intravenous fluid administration by the ambulance service prior to HEMS arrival, and a higher baseline heart rate (> 100 beats/minute) were associated with lower odds of post-PHEA critical hypertension.

Patients > 60 years old compose the largest proportion of major trauma patients in the UK [29]. Increasing age was associated with the incidence of critical hypertension in patients undergoing PHEA in this study; a finding supported by previous literature. However, the exact mechanisms for this are not well understood or studied [30, 31]. Laryngoscopy is a potent sympathetic stimulus - producing sudden increases in both heart rate and blood pressure (BP) [32]. Reduced arterial elasticity in the older patient impairs the ability to respond to sudden increases in cardiac output, and predisposes these patients to hypertension [33]. This, coupled with the slower distribution of anaesthetic agents in older people, may result in sub-optimal depth of anaesthesia at the time of intubation, and may account for an increased hypertensive response to laryngoscopy [34]. Anatomical changes, such as reduced cervical mobility, smaller mouth opening, and poor dentition may in addition contribute to the difficulty of intubation leading to prolonged attempts at laryngoscopy; propagating further sympathetic response [35].

Fentanyl is known to attenuate the hypertensive response to laryngoscopy [18, 23, 36]. This study has demonstrated that increased doses of fentanyl (up to 2mcg kg^− 1^) conveyed a protective effect against the outcome. These findings are consistent with the results from the recent FAKT trial, [17] in which patients that received a ketamine and rocuronium induction without fentanyl were significantly more likely to experience post-induction hypertension compared to placebo (55% fentanyl group vs. 69% placebo group) [17]. The optimal administration time of fentanyl to confer this benefit is not clear [36, 37]. By definition, rapid sequence induction (RSI) delivers medication with a 45-60-second interval before laryngoscopy. Such a short interval between drug administration and intubation may not be sufficient to attenuate the sympathetic response to laryngoscopy and contribute to the increased likelihood of hypertension following PHEA. In contrast, recent data in a prehospital cohort failed to demonstrate this effect, although doses were comparatively smaller [1.1mcg kg^− 1^ vs. 1.0-3.0mcg kg^− 1^] and used less frequently [17% vs. 67%] compared to this study [38]. A range of doses are associated with a lower risk of hypertension in the literature, from 1mcg kg^− 1^ to as high as 6mcg kg^− 1^ reported to almost eliminate any hypertensive response but often with greater occurrence of hypotension [39, 40]. The exact dosing to avoid physiological fluctuation is therefore likely to be a much more nuanced decision than a weight-based calculation alone, and likely includes variables such as age, injury pattern, haemodynamic status, and opiate-naivety.

The use of the GCS score to categorise severity of head injury (and general degree of consciousness) is well established, and the motor component is utilised in prehospital major trauma triage tools [41]. This study demonstrated that a higher GCS-M score, specifically GCS-M four or five, was associated with an increased risk of post-PHEA critical hypertension. Although GCS-M six did not reach significance, the lower-bound confidence interval of 0.99 may represent a type-2 error, and it is more plausible that this effect is present in patients with a GCS-M ≥ four (rather than just those with a GCS-M score of four or five). It is logical that patients with higher GCS-M scores, and therefore a higher level of consciousness, have greater odds of post-PHEA critical hypertension (through the mechanism of laryngeal stimulation) when controlled for all known confounders in this multi-variable model.

The traumatically injured brain is highly susceptible to even small changes in perfusion and oxygenation [42]. Increasing BP in TBI is likely to represent a critically injured brain attempting to maintain adequate cerebral blood flow [10]. With increasing BP comes increasing ICP if cerebral reactivity and autoregulation have been lost. Although this mechanism may be somewhat protective through maintenance of cerebral perfusion pressure, the net result may be damaging with exacerbation of vasogenic and cytotoxic cerebral oedema and ischaemia [15]. A clear association has been demonstrated between prehospital SBP and poor outcomes in TBI patients with a mortality inflection point at 180mmHg [10]. In this study, patients with a pre-PHEA SBP of 140-179mmHg were six times more likely to meet the outcome definition compared to normotensive patients. It is evident that the practice of PHEA in this cohort is exposing vulnerable patients, many of whom have suspected TBI, to critical hypertension (> 180mmHg). This may be harmful, worsen secondary brain injury, and contribute to mortality.

This study supports previous findings that multiple attempts at intubation are associated with post-PHEA hypertension [43, 44]. Despite a high reported first-pass success (FPS) rate in trauma PHEA, [45] in the small number of patients requiring more than one attempt, the adverse effect on BP increase is significant. Repeated attempts at laryngoscopy further provoke the sympathetic haemodynamic response, thus anticipation and preparation for a difficult airway, especially in patients most at risk, must be a fundamental consideration for the clinician performing PHEA. Whilst use of VL failed to reach significance in this study, the benefit of its use, particularly in achieving FPS has been recently published in the DEVICE (Direct versus Video Laryngoscope) randomised control trial [46]. With superior FPS rates when using VL as compared to DL (85% vs. 71%), utilising VL may decrease repeated laryngoscopy attempts and reduce the incidence of post-PHEA critical hypertension. In those at highest risk of post-PHEA critical hypertension, consideration should be given to the most experienced operator performing the intubation.

Administration of intravenous crystalloid by the ambulance service was associated with reduced odds of post-PHEA critical hypertension. UK ambulance service guidelines are prescriptive with regard to intravenous fluid in trauma patients; indicated only with evidence of impaired organ perfusion except in isolated head injury where a target SBP of 110mmHg is recommended if hypotension is present [47]. Whilst the physiological parameters at the time of the ambulance service attendance are not available, it is plausible that patients who received pre-HEMS intravenous fluid represent an initially hypotensive cohort, potentially with ongoing haemorrhage, unable to mount any additional sympathetic response. Similar ‘protection’ was found in patients with a pre-PHEA heart rate of > 100 beats/minute, which may reflect some patients with catecholamine depletion, also unable to intensify their sympathetic response. Tachycardia in trauma is due to a myriad of physiological and psychological factors, thus it is difficult to postulate with confidence why this isolated variable appears to demonstrate protection.

There has been a disproportionate publication within trauma academia exploring the impact of post-intubation *hypo*tension compared to *hyper*tension, in which the mortality implications are less certain. The recently published third edition Brain Trauma Foundation guidelines for prehospital TBI management advocate no increase in SBP > 150mmHg [13]. Whilst the outcome for critical hypertension in this study was selected as SBP > 180 mmHg, it is evident that this is not a binary cut off. Instead, there appears to be a trend of mortality with rising SBP from as low as 140mmHg [48].

Trauma patients, especially those with TBI, are susceptible to even subtle haemodynamic changes. Clinicians delivering PHEA to this high-risk patient group should be cognisant of the deleterious effects and transition from the simplistic approach of standardised induction drug regimes to a nuanced and tailored approach. The addition of invasive blood pressure (IBP) monitoring prior to induction may help to identify trends early and allow timely response to changes in physiology. In turn, IBP monitoring may offer reassurance to clinicians in the judicious administration of sympatholytic agents, such as fentanyl, for patients at most risk of critical hypertension. In the pursuit of avoiding hypotension, a practice of permissive hypertension may be just as harmful. Instead, the ultimate goal should be delivery of the most haemodynamically stable anaesthetic in this cohort of vulnerable patient, and further work is needed to explore the association between post-induction physiological derangement and patient centred outcomes.

### Limitations

Although a strength of this study is the reporting of the largest heterogeneous dataset of patients and clinicians evaluating physiological effects of PHEA in the UK, variations and evolution of practice may influence the results. *N = 204* patients were excluded for missing data, which may represent selection bias. However, this may also be explained by a cohort of profoundly hypotensive patients (in whom non-invasive blood pressure monitoring is often not capable of recording a value), as opposed to data missing-at-random. The retrospective nature and recognised difficulty of capturing data in the prehospital setting should be acknowledged. Conclusions should be considered as associative, not causative.

## Conclusion

Delivery of PHEA to seriously injured trauma patients risks haemodynamic fluctuation. In a UK population of adult trauma patients undergoing PHEA, 11.9% of patients experienced post-PHEA critical hypertension. Increasing age, pre-PHEA GCS motor score four and five, patients with a pre-PHEA SBP > 140mmHg, and more than intubation attempt were independently associated with post-PHEA critical hypertension.

## Data Availability

Restrictions apply to the availability of the datasets used and/or analysed during the current study and due to the sensitive nature and to protect confidentiality, are not publicly available.

## Abbreviations

BP: Blood pressure
CCP: Critical Care Paramedic
DBP: Diastolic blood pressure
DL: Direct laryngoscopy
EAAA: East Anglian Air Ambulance
EHAAT: Essex & Herts Air Ambulance Trust
FPS: First pass success
GCS: Glasgow Coma Scale
GCS-M: Glasgow Coma Scale motor
GCS-T: Glasgow Coma Scale total
HEMS: Helicopter Emergency Medical Service
HR: Heart rate
IBP: Invasive blood pressure
ICP: Intracranial pressure
IQR: Interquartile range
PEEP: Positive end expiratory pressure
PHEA: Prehospital emergency anaesthesia
RR: Respiratory rate
SBP: Systolic blood pressure
SD: Standard deviation
SOP: Standard operating practice
STROBE: Strengthening the reporting of observational studies in epidemiology
TBI: Traumatic brain injury
UK: United Kingdom
VL: Video laryngoscopy
